# Nutritional Characteristics, Health Impact, and Applications of Kefir

**DOI:** 10.3390/foods13071026

**Published:** 2024-03-27

**Authors:** Oladayo Emmanuel Apalowo, Grace Adeola Adegoye, Teresia Mbogori, Jayanthi Kandiah, Tolulope Mobolaji Obuotor

**Affiliations:** 1 Department of Food Science, Nutrition and Health Promotion, Mississippi State University, Starkville, MS 39762, USA; oea24@msstate.edu (O.E.A.); grace.adegoye@bsu.edu (G.A.A.); 2 Department of Nutrition and Health Science, Ball State University, Muncie, IN 47306, USA; tnmbogori@bsu.edu; 3 Department of Microbiology, Federal University of Agriculture, Abeokuta 110001, Nigeria

**Keywords:** kefir, micronutrient, diabetes, nutrients, probiotics

## Abstract

A global epidemiological shift has been observed in recent decades, characterized by an increase in age-related disorders, notably non-communicable chronic diseases, such as type 2 diabetes mellitus, cardiovascular and neurodegenerative diseases, and cancer. An appreciable causal link between changes in the gut microbiota and the onset of these maladies has been recognized, offering an avenue for effective management. Kefir, a probiotic-enriched fermented food, has gained significance in this setting due to its promising resource for the development of functional or value-added food formulations and its ability to reshape gut microbial composition. This has led to increasing commercial interest worldwide as it presents a natural beverage replete with health-promoting microbes and several bioactive compounds. Given the substantial role of the gut microbiota in human health and the etiology of several diseases, we conducted a comprehensive synthesis covering a total of 33 investigations involving experimental animal models, aimed to elucidate the regulatory influence of bioactive compounds present in kefir on gut microbiota and their potential in promoting optimal health. This review underscores the outstanding nutritional properties of kefir as a central repository of bioactive compounds encompassing micronutrients and amino acids and delineates their regulatory effects at deficient, adequate, and supra-nutritional intakes on the gut microbiota and their broader physiological consequences. Furthermore, an investigation of putative mechanisms that govern the regulatory effects of kefir on the gut microbiota and its connections with various human diseases was discussed, along with potential applications in the food industry.

## 1. Introduction

The global population of individuals aged 65 and older is anticipated to increase significantly, from 10% in 2022 to 16% by 2050, reflecting an ongoing and rapid global aging phenomenon [1]. This accelerated trend in life expectancy presents substantial challenges to both public health and socioeconomic systems. As individuals age, they become more susceptible to non-communicable diseases such as type 2 diabetes (T2D), cardiovascular illnesses, cancer, and neurodegenerative conditions [2], necessitating continuous monitoring and medical intervention.

Several interventions including dietary changes, physical activity, metformin, nicotinamide adenine dinucleotide (NAD^+^) precursors, sirtuin agonists, senescence-associated secretory phenotype (SASP) inhibitors, and senolytics are currently being developed to target underlying aging mechanisms with the potential to impact multiple fundamental aging pathways [3]. These all have a significant impact on the makeup of the gut microbiome, as do the changes in lifestyle choices that follow, such as a reduction in the quality of food and physical activity and an increase in drug use, which have been linked to the onset of age-related disorders [4,5,6]. Thus, one of the most important treatment approaches might be to alter the microbiome through food to slow down the physiological deterioration that comes with age [7]. The consumption of dietary components may either feed or restrict the growth of specific members of the gut microbial population, making nutrition a significant regulator of the microbiome [8].

Kefir is a fermented milk beverage produced by the synergistic action of bacteria and yeasts contained in kefir grains that have an inert matrix of polysaccharides and proteins and are home to a diverse range of microorganisms, including lactic acid bacteria (LAB), acetic acid bacteria, and yeasts [9,10]. The intricate interactions of these multiple microbes, as well as several bioactive substances produced by their metabolic processes, contribute to kefir’s reputation as a natural probiotic [9,11,12,13,14]. The microbial composition of kefir can vary based on factors like geographical origin, fermentation duration, substrate, and processing methods, yet kefir grains consistently maintain a relatively stable and distinctive microbiota, often characterized by the prevalence of specific *Lactobacillus* species [15].

The interplay between diet and gut microbiota has a major impact on species abundance, diversity, and overall influence on human health [16]. As a result, an in-depth investigation of the impacts of kefir’s bioactive compounds on the gut microbiota is required, which will contribute to a better understanding of the underlying processes driving kefir’s role in human health. In this perspective, we discussed 33 studies from 2012 to 2024 that investigate the impact of kefir’s bioactive compounds on the gut microbiota and metabolic physiology under deficient and supplemented conditions and, in addition, extended this discussion to age-related diseases.

## 2. Gut Microbiota Changes with Age

Microorganisms that make up the intestinal microbiota may exist in two states: balanced or unbalanced. The microbiota in the first situation, known as eubiosis, is flexible enough to maintain its equilibrium by tolerating minor changes in the environment, the diet, or the water drunk. However, instances of significant changes, such as the translocation or expansion of a particular bacterial group, colonization by pathogenic bacteria, the use of antibiotics, and alterations in lifestyle, result in imbalance or dysbiosis [17,18]. Since the microbiota is primarily governed by physiology, the relative abundance of some microbes is affected by age-related changes in intestinal physiology with respect to the host’s diet, lifestyle, and medications [19,20].

Mucin, for example, functions as a protective barrier in the gastrointestinal system, preventing direct contact between microbes and epithelial cells. Nonetheless, in mice, mucin production declines with age, resulting in a thinner and less uniform mucus coating. Different microbial strains, such as those in the *Clostridiaceae*, *Akkermansiaceae*, *Bifidobacteriaceae*, and *Bacteroidaceae* families, all display age-related alterations and use mucin as a nutrient [21]. Although the role of mucin-metabolizing microbes like *Akkermansia muciniphila*, *Bacteriodes fragilis*, *Bacteroides vulgatus*, *Bifidobacterium* spp., and *Prevotella* spp. in mucin layer degradation with age remains unclear, *Bacteroides* spp. and *A. muciniphila* increase in centenarians, hence implying potential benefits [22]. As a result, probiotic-enriched foods or supplementation with certain strains may improve age-related mucin loss, as well as good benefits on health, immunity, and lifespan [23]. Increased amounts of short-chain fatty acids (SCFAs) produced as byproducts by the gut microbiota during the fermentation of partly and non-digestible polysaccharides resulted in a decrease in colon pH, which contributed to the enhancement of the intestinal barrier [24,25].

However, as the protective mucin layer in the digestive system deteriorates, microbes that typically would not contact the epithelial layer can now potentially trigger inflammatory responses [6,26]. Consequently, aging, as well as the chronic health and metabolic disorders that come with it, is marked by a rise in low-grade, chronic inflammation [6]. Therefore, enhancing the preservation of intestinal physiology in model organisms can mitigate age-related alterations, ultimately minimizing microbial dysbiosis and extending lifespan [27].

The intestinal microbiota has an impact on the operation of several organs, including the heart, brain, liver, pancreas, and gut, and its regulation may be a crucial step in the treatment of illnesses and the preservation of health since the intestinal microbiota also plays a role in the development and maturation of organs and physiological systems [28,29]. Increased intestinal permeability relates to an imbalance between Firmicutes and Bacteroidetes, which permits bacterial byproducts to penetrate and induce inflammatory reactions associated with diabetes and other metabolic disease [30]. LPS from Gram-negative bacteria induces insulin resistance and impairs insulin signaling in a variety of organs, including muscle, adipose tissue, liver, and the hypothalamus, via activation of NF-kB and JNK pathways. Additionally, LPS activates toll-like receptors, triggering the production of inflammatory cytokines, thereby activating the immune system [31,32].

## 3. Kefir: Composition and Nutritional Profile

### 3.1. Occurrence

Kefir is a fermented beverage made from kefir grains and milk or water that is acidic, frothy, and low in alcohol [33,34]. Its origins may be found in the Caucasus, Eastern Europe, and the Balkans, and due to its beneficial health effects, its consumption has spread worldwide throughout time [35]. People in nations like the United States of America, Japan, France, and Brazil have become accustomed to drinking this sour, viscous beverage [36]. The distinctive quality of its starter, the kefir grains, is how kefir differs from other fermented foods.

Kefir grains are irregularly shaped and lobed, ranging from white to light yellow [37]. They are 1 to 4 cm long and resemble miniature cauliflower florets. Lactic acid bacteria (LAB), yeasts, and acetic acid bacteria (AAB) coexist in symbiotic association inside the natural matrix of exopolysaccharides (EPS), kefiran, and proteins that make up this gelatinous and slimy structure [34]. The use of kefir beverages has been linked to significant health advantages, including improved lactose digestion, anti-carcinogenic, anti-hypertensive, and anti-diabetic effects [38,39,40]. The kefir bacteria, their interactions, and their metabolic products throughout the fermentation process are responsible for all these health-promoting qualities [34].

### 3.2. Microbial Diversity

Lactic acid bacteria, acetic bacteria, yeasts, and fungi are the most prevalent microbial species in kefir grains, among other complicated microbial species [41], and have been divided into four groups: homo- and heterofermentative as well as lactose- and non-lactose-assimilating yeasts [42]. *Lactobacillus kefiranofaciens*, *Lacticaseibacillus paracasei* (also known as *Lactobacillus paracasei*), *Lactiplantibacillus plantarum* (also known as *Lactobacillus plantarum*), *Lactobacillus acidophilus*, and *Lactobacillus delbrueckii* subsp. *bulgaricus* are the most prevalent bacterial species found in kefir grains, while the main yeast species found in kefir are *Saccharomyces cerevisiae*, *S. unisporus*, *Candida kefyr*, and *Kluyveromyces marxianus* subsp. *marxianus* [35].

According to the geographic origin of the kefir grains, which is inextricably linked to the climatic conditions, the microbiota of the kefir grains may vary [43]. In actuality, the ratio of kefir grains to substrate, fermentation period, temperature, and degree of agitation are all factors that might affect the microbiota composition and nutritional content of kefir [44,45]. Although some significant *Lactobacillus* species always occur due to their probiotic strain-specific capabilities, it is acknowledged that this microbial diversity is responsible for the physicochemical characteristics and biological activities of each kefir [44,46].

### 3.3. Macronutrients and Micronutrients

Kefir contains high concentrations of essential vitamins (>20%) such as thiamin (B1), riboflavin (B2), pantothenic acid (B5), folic acid (B9), ascorbic acid (C), retinol (A), and phylloquinone (K); essential amino acids (70–376 mg/100 g) like serine, threonine, alanine, lysine, valine, isoleucine, methionine, phenylalanine, and tryptophan; macro elements such as potassium (1.65%), calcium (0.86%), phosphorus (1.45%), and magnesium (0.30%); and microelements including zinc (92.7), copper (7.32 mg/kg), iron (20.3 mg/kg), manganese (13.0 mg/kg), cobalt (0.16 mg/kg), and molybdenum (0.33 mg/kg), respectively [47,48,49,50,51]. Recently, GCMS analysis showed the presence of alkaloids, phenols, esters, fatty acids, steroids (cholesterol and ergosterol), polyalkenes, heterocyclic compounds, and aromatic aldehydes [52].

**Figure 1:**
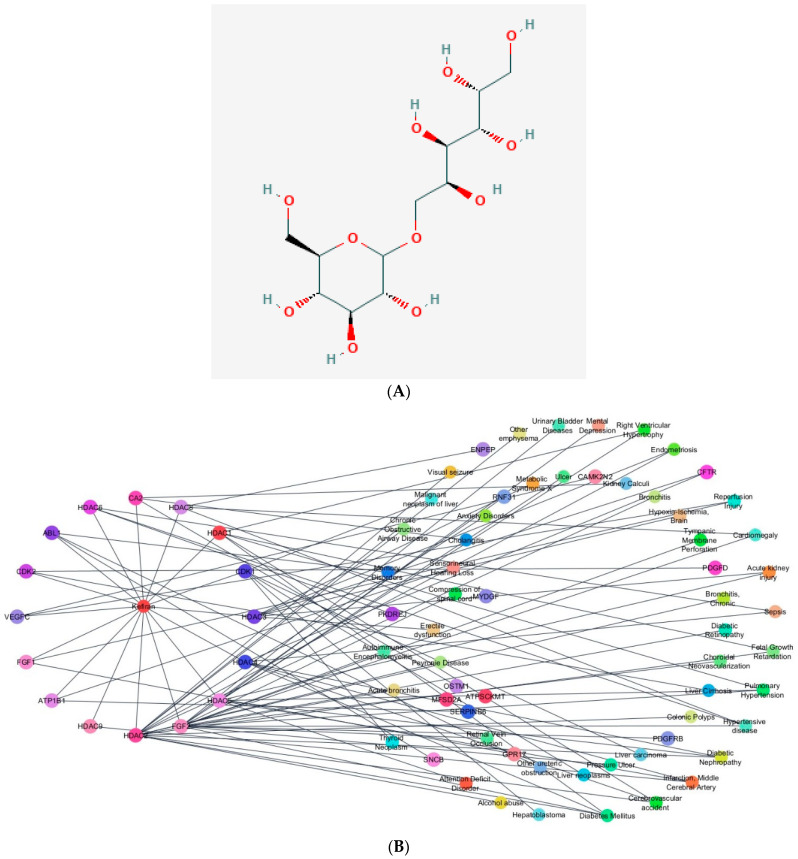
(**A**) Two-dimensional (2D) structure of Kefiran (PubChem CID 90908346). (**B**) Potential roles of Kefiran on different disease phenotypes using network analysis. Kefiran–protein interaction generated from BindingDB [56] and gene–disease association curated from DisGeNet [57]. Integrated biomolecular interaction was created using Cytoscape [58] and STRING.db [59]. Target genes are listed as follows: Abl1, non-receptor tyrosine kinase; ca2, carbonic anhydrase II; cdk2, cyclin-dependent kinase 2; cdk1, cyclin-dependent kinase; fgf1, fibroblast growth factor 1; fgf2, fibroblast growth factor 2; hdac, histone deacetylase; atp1b1, ATPase Na^+^/K^+^ transporting subunit beta 1; vegfc, vascular endothelial growth factor C.

Many of these compounds have different absorption rates in the brain and may enter as rapidly as glucose to affect metabolic processes. For example, since essential amino acids cannot be synthesized by the brain, they must be supplied from protein breakdown and diet. Hence, these components are essential for controlling energy balance, serving as precursors to neurotransmitters synthesized in the brain, supporting metabolic activities, enhancing immunomodulation, and fostering homeostasis [53]. Kefiran, a microbial polysaccharide from kefir grains, aids in the mental recovery of individuals with severe traumatic brain injuries and lengthens the healthy lifetime of the elderly [54,55]. Using database mining approaches, we revealed that kefiran binds to different protein targets that may partake in several molecular events (Figure 1). Hence, kefir is suggested to exert control of organism homeostasis through a direct impact on the gut–brain axis [17].

Numerous studies have been conducted to investigate the modulatory effects of these bioactive substances on the gut microbiota in experimental animal models, as well as the resulting metabolic implications. Table 1 summarizes pertinent information about the experimental models, designs used, and results obtained from the research.

## 4. Kefir: Implications for Healthy Aging

### 4.1. Type 2 Diabetes

T2D is a complicated chronic disorder that puts patients at risk for long-term macro- and microvascular consequences due to deficiencies in insulin secretion, glucose metabolism, or both [43]. Chronic low-grade inflammation is correlated with the onset of T2D. An unbalanced intestinal microbiota, which is promoted by changes in intestinal permeability caused by LPS-induced endotoxemia, favors the development of this inflammation, resulting in systemic insulin resistance and the subsequent onset of metabolic syndrome and T2D [17].

A study using high-fat diet-fed mice found that *Lactobacillus mali* APS1 produced from kefir grains lowered blood glucose and HOMA index while increasing glucagon-like peptide (GLP-1) and butyrate levels [90,91]. Lowering the HOMA index indicates better glycemic control while increasing GLP-1 implies appetite modulation and possibly protection for insulin-producing pancreatic beta cells, which are essential for controlling blood sugar levels [92]. Another study on Wistar rats with monosodium glutamate-induced metabolic syndrome revealed that whole milk kefir consumption for 10 weeks reduced insulin resistance. This improvement was linked to the calcium content in kefir and the bioactive compounds generated during fermentation. Additionally, kefir enhanced glucose uptake by muscle cells, further reducing insulin resistance [93].

Patients with type 2 diabetes have a microbiota that is characterized by a reduction in butyrate-producing bacteria, a moderate dysbiosis, an environment that is pro-inflammatory, a decrease in the expression of genes involved in vitamin synthesis, an increase in serum LPS levels, and an increased intestinal permeability [94]. However, the gut microbiota contributes to energy generation via anaerobic digestion of food components, which results in SCFA such as acetate, propionate, and butyrate. Notably, butyrate, a major source of energy for colonocytes and the main byproduct of SCFA fermentation, is known to regulate body weight and improve insulin sensitivity by boosting GLP-1 secretion via GPR-mediated signaling and decreasing adipocyte inflammation [90].

A further beneficial effect of Kefir on T2D was discovered in a study involving 60 diabetic individuals aged 35 to 65. The patients were split into two groups, one receiving kefir containing *L. casei*, *L. acidophilus*, and *B. lactis* and the other receiving conventional fermented milk containing *Streptococcus thermophiles* and *Lactobacillus bulgaricus*. For eight weeks, each group drank 600 mL of their assigned treatment beverage each day. Following the intervention, individuals receiving kefir had lower levels of glycated hemoglobin and fasting glucose than the group receiving the other fermented beverage although no significant changes in serum triglyceride, TC, LDL-c, and HDL-c [95]. Currently, there are only a few clinical studies linking kefir intake with gut microbiota composition in healthy and disease conditions (Table 2).

### 4.2. Cardiovascular Health

Dyslipidemia, a known risk factor for cardiovascular disease, causes decreased diversity in the gut microbiota, rendering people more vulnerable to dysbiosis. This increased susceptibility causes inflammation and alterations in intestinal permeability, resulting in negative health effects for the host. SCFAs contribute to energy generation, lipogenesis, gluconeogenesis, and cholesterol synthesis, in addition to primary bile acids capable of binding to the farnesoid X receptor, which is an important protein in the etiology of obesity [101,102]. However, kefir blocked intestinal lipid uptake in obese mice through the reduction in hepatic and serum triglycerides, total cholesterol, and LDL-c as well as reduced expression of genes linked to adipogenesis, lipogenesis, and proinflammatory cytokines in epididymal fat [103].

Despite the little exploration of its biological profile, several biologically active peptides are produced by the symbiotic metabolic interactions between different bacterial and yeast species in kefir, including ACE-inhibitory peptides that block the angiotensin-converting enzyme (ACE), preventing the conversion of angiotensin I to the vasoconstrictor angiotensin II [104]. This inhibits the production of aldosterone, which typically increases serum sodium levels and elevates blood pressure, while also impacting bradykinin, a vasodilatory hormone, resulting in decreased blood pressure [50,93].

In addition, the discovery of 16 peptides released from caseins, including two (sequences PYVRYL and LVYPFTGPIPN) exhibiting strong ACE-inhibitory capabilities, shows that commercial kefir made from caprine milk demonstrates considerable angiotensin-converting enzyme (ACE)-inhibitory activity [105]. These findings imply that kefir may harbor a wide variety of bioactive chemicals with the ability to work independently or synergistically, making it a suitable therapeutic option or adjunct in the treatment of hypertension.

Furthermore, a soluble non-bacterial fraction of kefir reduced cardiac hypertrophy in spontaneously hypertensive rats (SHRs), possibly through ACE inhibition and lowering the TNF-α-to-IL10 ratio and improved baroreflex sensitivity in hypertensive rats. It also decreased mean arterial pressure (MAP) and heart rate (HR) [39]. Kefir was also shown to increase baroreflex sensitivity (BRS) in SHR and to reduce cardiac autonomic control impairment when taken regularly in modest doses [106].

In another separate study, a 60-day kefir treatment improved endothelial function in SHR, which was attributed to the partial restoration of the ROS/NO balance and the recruitment of endothelial progenitor cells, both of which contributed to the improvement of the endothelial architecture [107]. Also, administration of pitched (traditional) kefir (350 g of kefir/day) for 4 weeks in adult males reduced high LDL-c, ICAM-1, VCAM-1, IL-8, TNF- α, and CRP, indicating the metabolic impact of kefir intake [108].

**Figure 2:**
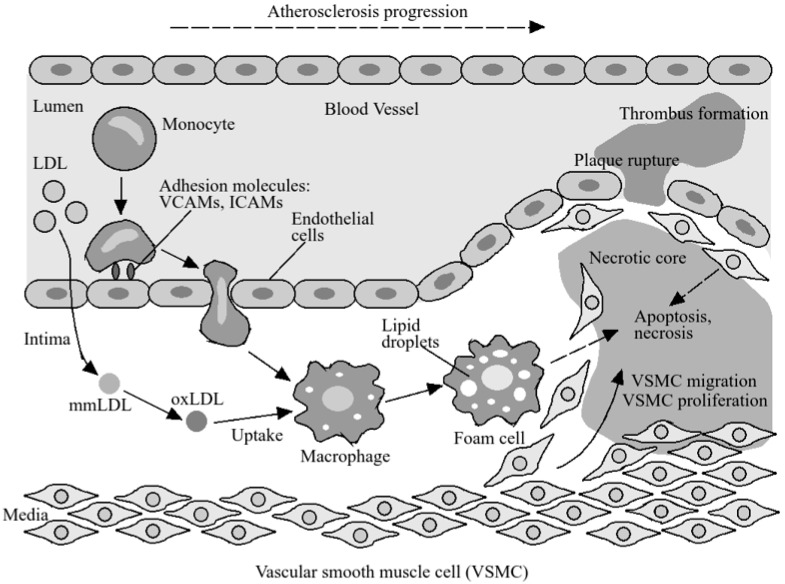
Atherosclerotic plaque development initiated by lipid retention, oxidation, and modification.

As shown in Figure 2 [109], LDL oxidation is regarded as the primary cause of atherosclerotic plaque development and a substantial contributor to proinflammatory responses in the subendothelial area [110]. The involvement of HDL in lipid distribution, enabling the absorption and movement of cholesterol deposited in atherosclerotic plaque foam cells back to the liver and bile (cholesterol reverse transport), highlights the anti-atherogenic and anti-inflammatory effects of HDL [111,112], NO-promoting effects, and inhibition of TNF-α-induced endothelial cell apoptosis [113,114].

HDL inhibits the cytotoxic impact of oxidized LDL on the vascular endothelium that triggers the atherogenesis process, reducing inflammation by inhibiting the expression of adhesion molecules on the endothelium surface, such as P-selectin, E-selectin, ICAM-1, and VCAM-1 leading to reduced T-cell and monocyte adherence to the vascular endothelium, hence restricting their movement to atheromatous focus. Pro-inflammatory processes are initiated when soluble forms of endothelial adhesion molecules, sP-selectin, sE-selectin, sICAM-1, and sVCAM-1 are released into the bloodstream from the surface of active endotheliocytes [115]. The significant reduction in lipid profile parameters including LDL-c, cell adhesion molecules ICAM-1 and VCAM-1, as well as proinflammatory cytokines such as IL-8, TNF-α, and IL-17a after kefir intake reveals its atheroprotective effect, protecting against cardiovascular disease.

### 4.3. Cognitive Function and Alzheimer’s Disease

A wealth of data shows that diverse stimuli affect the intestinal mucosa, influencing gut integrity via the hypothalamic–pituitary–adrenal axis [116,117]. This link extends to neurodegenerative conditions and gut dysbiosis, with gut microbiota-derived molecules impacting the blood–brain barrier and brain function [118]. The development of Alzheimer’s disease is linked to an imbalance in the microbiota–gut–brain axis, which may be aggravated by a lack of probiotic bacteria owing to an imbalanced diet [119]. Microglial maturation and function, blood–brain barrier construction and stability, myelination, and neurogenesis, among other neurodevelopmental processes, have all been discovered to be influenced by gut microbiota and contribute to neurological health [17,120].

In an experimental study involving an animal model exposed to nicotine-induced stress, Noori et al. [121] investigated the potential therapeutic effects of fermented kefir made from both soy and cow’s milk on depression, anxiety, and cognitive impairment. A battery of tests, including the elevated plus maze (EPM) for anxiety assessment, the open field test (OFT) to evaluate locomotor activity and anxiety, and the forced swim test (FST) for measuring depression severity, was employed. Throughout the treatment period, a significant improvement in anxiety reduction, depression severity, and cognitive performance was observed and is thought to be related to kefir’s high tryptophan content, which is a precursor to serotonin, a neuromodulator that plays an important role in fostering neuroplasticity and neuronal growth associated with depression [122].

Similarly, in an animal model mirroring human depression induced by six weeks of exposure to seven stressors, mice supplemented with *Lactobacillus kefiranofaciens* ZW3, sourced from kefir, displayed increased activity, a greater preference for sucrose, and reduced risk of constipation, a condition associated with depression. This supplementation also led to improved tryptophan metabolism, elevated anti-inflammatory cytokines, decreased pro-inflammatory cytokines, and notable changes in the gut microbiota composition, including increases in Actinobacteria, *Bacteroides*, *Lachnospiraceae*, *Coriobacteriaceae*, *Bifidobacteriaceae*, and *Akkermansia* and decrease in Proteobacteria [123].

Tryptophan stimulates SIRT1, PGC-1α, and FOXO1 in colons and brains, reducing aging-related defects via AMPK/SIRT1/PGC-1α and PXR/TLR4/NF-κB pathway regulation [124]. SIRT1, which is dependent on NAD^+^, promotes longevity by controlling energy metabolism, mitochondrial function, and anti-aging via AMPK [125,126]. SIRT1 reduces oxidative damage, inflammation, and apoptosis by upregulating the expression of PGC-1α, FOXO1, NF-κB, and Bax [127,128]. AMPK/SIRT1 promotes CREB/BDNF pathway expression, reducing cognitive impairment associated with aging [129]. Indoles, a tryptophan metabolite, improve gut barrier function by suppressing TLR4/NF-κB through PXR [130].

Oxidative stress is important to the pathophysiology of Alzheimer’s disease (AD), impacting the brain more deeply due to mitochondrial malfunction, increased metal levels, inflammation, and β-amyloid peptides [131]. An in vivo study utilizing aged mice reveals that probiotics fermentation technology (PFT) containing specific microbes namely *Lactobacillus kefiri P-IF*, *L. kefiri P-B1*, *Kazachstania turicensis*, *Kazachstania unispora*, and *Kluyveromyces marxianus* reduces age-related oxidative stress. A six-week oral daily dosage of 2 mg/kg body weight PFT suppresses oxygen radical generation, enhances GSH and total antioxidant capacity, and reduces NO and MDA levels, restoring age-related oxidative alterations to levels comparable to those seen in young, untreated mice [132].

Furthermore, in a study using a fly model of AD, supplementing their diet with kefir made from grains containing *Lactobacillus* species (*L. kefiranofaciens*, *L. kefiri*, *Acetobacter fabarum*, *L. lactis*, and *Rickettsiales*) resulted in significant improvements. This included a 1.6-fold increase in survival rates, a 2-fold enhancement in climbing ability, and reduced severity of brain vacuolar lesions, all indicating a positive impact on the neurodegenerative phenotype of the flies [133]. Kefir-fed animals had a unique gut microbiota composition, which is thought to have a favorable effect on the gut–brain axis. Microbiome analysis suggests that kefir may boost the synthesis of gamma-aminobutyric acid (GABA), an inhibitory neurotransmitter. This action is thought to be mediated by the conversion of 2-oxoglutarate to glutamate, which may be aided by *Lactobacillus reuteri*. The manipulation of the intestinal microbiota by kefir results in an increased presence of *Lactobacillus reuteri*, a bacterial species renowned for its positive effects on the host’s immunological and metabolic systems [134].

In humans, kefir was shown to improve the symptoms of AD in human studies. Kefir consumption by patients with probable AD for 90 days showed substantial cognitive improvements, including higher memory test scores, enhanced visual–spatial and abstraction abilities, better executive, and language functions, and improved Mini-Mental State Examination (MMSE) scores, which were accompanied by reduced serum TNF-α, IL-12p70, IL-8, enhanced mitochondrial membrane potential, and reduced intracellular ROS in erythrocytes [135].

### 4.4. Cancer

Since cancer cells proliferate rapidly and are resistant to apoptosis, with a strong relationship to poor dietary habits, it is critical to investigate dietary components, particularly probiotics such as kefir as a possible therapeutic option for mitigating cancer cell growth [136]. During neoplastic growth, tumor cells evade the immune response, and probiotic bacteria have been found to promote immune system effector activities in co-cultures with peripheral blood mononuclear cells (PBMCs), as evidenced by cytokine profiles [137,138].

Previous research has demonstrated the involvement of kefir in the anti-tumor process in many malignancies, such as breast cancer [139,140], leukemia [141,142], skin cancer [143], gastric cancer [144,145], colon cancer [146,147], and sarcomas [148,149].

In a study to examine the effects of kefir on colorectal cancer (CRC) via gut microbiota composition regulation using internally transcribed spacer 2 (ITS2) and 16S rRNA high-throughput sequencing on azoxymethane/dextran sulfate sodium (AOM/DSS)-induced CRC mouse model, kefir supplementation decreased the ratios of Firmicutes/Bacteriodetes and Ascomycota/Basidiomycota ratio, as well as the relative abundance of pathogenic bacteria *Clostridium* sensu stricto, *Aspergillus* and *Talaromyces*. This was accompanied by a decrease in oncocyte proliferation indicator (Ki67, NF-κB, and β-catenin) and immunity regulators (TNF-α, IL-6, and IL-17a) while the relative abundance of probiotics increased [24].

Further study showed that administration of *Lactobacillus kefiranofaciens* JKSP109 (LK) and *Saccharomyces cerevisiae* JKSP39 (SC) from Tibetan kefir grain and their combination on an AOM/DSS-induced mouse model of CRC led to an increased expression of TUNEL-positive tumor epithelial cells and the content of short-chain fatty acids in fecal samples, as well as increase in body weights while disease activity index, tumor multiplicity, and proinflammatory cytokines were reduced [150]. Likewise, several similar studies have reported the anticancer properties of kefir including its anti-tumor effect via the promotion of tumor immunotherapy by modulating the gut microbiota composition [151], regulation of intestinal inflammation, and subsequent reduction of DMH-induced CRC in Wistar rat’s offspring programmed for adulthood through neonatal overfeeding [152].

Kefir has been found to exert its anticancer effects via multiple mechanisms [136]. Kefir contains bioactive peptides that stimulate macrophage activation, phagocytosis, and NO generation, resulting in increased TNF-α, IL-5, IL-6, IL-1β, and IL-12 levels, leading to increased IgA secretion and inhibition of inflammatory responses via decreased IL-8 secretion [40,136,153]. Kefir consumption also controls apoptosis by lowering TGF-α, TGF-β, and Bcl2 secretion while boosting bax secretion. Kefir’s active peptides trigger ROS-mediated apoptosis and stimulate Ca^2+^/Mg^2+^-dependent endonucleases for DNA cleavage [154]. An anti-proliferative impact on malignant cells is triggered by low TGF-α and TGF-β secretion, while kefir’s sphingomyelins increase IFN-β release, which acts as an anti-proliferative cytokine [155,156].

## 5. Applications in Food Product Development

Kefir, known for its diverse beneficial microorganisms and bioactive compounds, offers a range of health benefits in dairy products, although milk-related hypersensitivities pose challenges. In response, fermented non-dairy beverages are gaining popularity, prompting scientific exploration of kefir adaptation onto non-dairy substrates like fruits, vegetables, and molasses for diverse fermentation bioprocesses [157].

A recent study examined how the amount of kefir grains and fermentation time affected the composition, sensory aspects, and color of a probiotic beverage. The study found substantial impacts, such as a drop in sugar content, and an increase in acidity, total phenols, carbon dioxide, and organic acids. The study also discovered that fermentation altered sensory qualities such as color, brightness, chroma values, density, antioxidant activity, citric acid, and hue values, with differences related to the quantity of kefir grains inoculum and fermentation period [158].

Natural materials are increasingly being used in the food and packaging industries to provide ecologically friendly and biodegradable packaging solutions [159]. A recent study investigated the effects of kefir on the quality of gelatine-based edible films. The findings revealed that, while the thickness of the films remained constant, density rose and hydrophilic characteristics improved. Although kefir improved surface morphology, excessive application resulted in hazy formations and a minor loss in mechanical characteristics. The inclusion of kefir enhanced the greenness, yellowness, and opacity of the films. This suggests that kefir could be a more environmentally sustainable alternative to petrochemical packaging, as it showed no growth of harmful microorganisms over a 10-day period, highlighting potential benefits for both environmental sustainability and human health [160].

Besides food product development, a recent study examined the potential of kefiran, a biopolymer, for tissue engineering and regenerative medicine applications [161]. Extracted kefiran was used to produce scaffolds by cryogelation and freeze-drying, with molecular structures validated by proton NMR and FTIR spectra. The results indicated a high molecular weight, acceptable rheological properties, and scaffold traits like porosity and wall thickness. The kefiran extracts and scaffolds had no cytotoxic impact on L929 cells and showed no significant differences in cytocompatibility, which altogether positions them as potential options in tissue engineering and regenerative medicine.

Additionally, while traditional kefir made from natural grain-based kefir is reported to elicit health-benefiting properties, commercial kefirs made of defined mixtures of microorganisms are emerging with different functional effects. Due to the complexities of using grains and the resulting limitations on product shelf-life, modern commercial kefirs employ artificial microbial blends. Commercial kefir production now involves various starter culture producers and dairy product manufacturers. Research indicates that commercial kefirs differ significantly from traditional grain-based kefir in microbial composition and metabolite characteristics, which therefore necessitates further investigation on their potential in the food industry and functional effects on consumers [157,162].

## 6. Perspectives

The manipulation of the intestinal microbiota has emerged as a promising strategy for both disease prevention and therapeutic intervention, and the utilization of fermented foods possessing probiotic properties offers a nutritional approach as an alternative to synthesized drugs. Kefir, distinguished by its established safety profile in both animal and human, cost-effectiveness, ease of preparation, and microbiological composition enriched with bioactive compounds, metabolites, and peptides, stands out as a potential functional food with substantial health benefits.

Kefir contains bioactive compounds and unique peptides; it harbors specific bacterial strains known to orchestrate shifts in the composition of gut microbiota, alleviate low-grade inflammation, and promote optimal health. These result in a wide range of health advantages that may lessen the growing prevalence of these age-related disorders. Additionally, the modern food market is seeing the rise of novel functional foods, fueled by the remarkable therapeutic capabilities of kefir grains. This has resulted in a greater demand for healthier and more sustainable food items infused with kefir and its value-added derivatives. Furthermore, specialists in nutrition and food science are expressing a strong interest in extending research disciplines to explore the therapeutic qualities of kefir.

However, further research is necessary to exploit the advantages that highlight kefir’s potential in healthy aging, specifically to (1) comprehend the precise microorganisms orchestrating its beneficial effects, their complex molecular interactions with other bioactive compounds within the gut (whether as synbiotics or postbiotics), and how they impact the gut–brain axis; and (2) perform controlled human intervention trials that might enable a more robust approach to elucidating the specific functional mechanisms by which kefir exerts its biological benefits, particularly in the context of promoting healthy aging.

## Figures and Tables

**Table 1:** Roles of kefir bioactive constituents on gut microbiota and physiological parameters in both health and disease conditions.

Bioactive Compounds	Research Design	Experimental Model	Methods	Gut Microbiota Alteration	Physiological Effect	Ref.
Retinol (vitamin A)	Supplementation: 5000 IU retinyl acetate for 10 days	7-week-old male 57BL/6Cnc mice with ulcerative colitis	16S rRNA gene sequencing; qPCR; TRACE 1310-ISQ LT GC-MS system	Abundant gut microbial diversity and flora composition; decrease in *Bacteroides*, *Parabacteroides*, *Escherichia/Shigella*, *Klebsiella*, *Oscillibacter*, *Pseudolavonifractor*, *Clostridium* sensu stricto, *Butyrimimonas*, *Mucispirllum*, and *Clostridium* XIVb; increased abundance of *Akkermansia*, *Lactobacillus*, *Prevotella*, and *Aerococcus*	Significant increase in SCFAs: acetic acid, butyric acid, propionic acid, valeric acid, isobutyric acid, and isovaleric acid except caproic acid (*p* < 0.05); significant increase in mRNA expression of *Muc1*, *Muc4*, *ZO-1*, *occludin*, and *IL-10* (*p* < 0.05); mRNA expression of *IL-6*, *IL-1β*, and *TNF-α* and serum levels of LPS, TNF-α, IL-6, and IL-1β significantly reduced (*p* < 0.05)	[60]
	Supplementation: 25 IU/g for 16 weeks	3-week-old male C57BL/6J mice with neuronal and cognitive alterations	16S rRNA gene sequencing; qPCR; NMR; HPLC; LC-ESI-MS/MS; Varian 3500 GC flame-ionization system	Increased the abundance of *Lachnospiraceae*, *Porphyromonadaceae*, *Mycoplasmataceae*, and *Subdoligranulum*; decrease in RC9	Significant reduction in weight gain, fat mass, leptin, and insulin without any significant change in SCFA and BCFA (*p* < 0.05); similarly, no significant effect on the expression of genes coding for proteins involved in GC signaling, namely GC receptors, mineralocorticoid receptors, and *11β-Hsd1*enzyme, responsible for the local production of active GC; prevented recognition memory deficits	[61]
	Deficiency VA Knockout model	7-week-old male C57BL/6J mice with altered metabolism	16S rRNA gene sequencing; qPCR; proton (^1^H) NMR	Significantly lower abundance of Bacteroidetes, Bacteroidia, *Pseudomonadaceae*, *Clostridium*_XVIII, *Roseburia*, *Blautia*, *Pseudomonas*, *Parabacteroides*; Increased Firmicutes/Bacteriodes ratio, *Johnsonella*, and *Staphylococcaceae* (*p* < 0.05)	Increased acetate, significantly higher levels of lactate and glucose in serum and liver; decreased butyrate levels; glucose clearance was slower; increased level of carbohydrate, lower lipid, and amino acids (BCAA) levels; hyperglycemic state induction and increased protein metabolism	[62]
	Deficiency: VA < 120 IU/kg for 45 weeks	8-week-old male C57BL/6J APP/PS1 transgenic mice with Alzheimer’s disease	16S rRNA gene sequencing; RP-HPLC; qPCR	Enrichment in pro-AD pro-AD Clostridia and decreased abundance of anti-AD *Lactobacillus*	VA deprivation resulted in increased deposition of Aβ plaque (Aβ40 and Aβ42) and increased expression of BACE1 and p-Tau (*p* < 0.05); downregulation of GABA_Aα2_, GABA_B1b_, and BDNF in the cortex and hippocampus (*p* < 0.05); decreased mRNA expression of *RARγ*, *RALDH1*, *RXRα*, *RXRβ*, *RXRγ*, and *CYP26B1* genes in the cortex (*p* < 0.05)	[63]
	Deficiency: VA 300 IU/Kg for 4 weeks	3-week-old female Sprague–Dawley rats; impairment of colonic epithelial barrier integrity	16s rRNA gene sequencing; qRT-PCR; HPLC; GC-MS	Predominant phyla: Firmicutes (60.25%), Verrucomicrobiota (14.47%), Bacteroidota (13.74%), Proteobacteria (9.26%), Actinobacteriota (1.14%), and Desulfobacterota (0.88%); Family: decreased relative abundances of *Peptostreptococcaceae*, *Erysipelotrichaceae*, *Coriobacteriaceae*, *Eggerthellaceae*, and *Staphylococcaceae* while there is an increased relative abundance of *Bacteroidaceae*, *Streptococcaceae*, *Butyricicoccaceae*, and *Actinomycetaceae* (*p* < 0.05) Genus: decreased relative abundances of *Romboutsia*, *Collinsella*, *norank_F_Erysipelotrichaceae*, and *Allobaculum* while there is an increased relative abundance of *Bacteroides*, *norank_f_Oscillospiraceae*, *Lachnospiraceae*_NK4A136_group, *Colidextribacter*, and *Streptococcus* (*p* < 0.05)	Reduced weight (*p* < 0.001); shorter lengths and looser fibrils of desmosome junctions (*p* < 0.05); increased level of DAO (*p* < 0.05),evidence of a leaky gut; decreased expression of CEACAM1; increased HDAC1 and HDAC3 expression (*p* < 0.05)	[64]
Folic acid (vitamin B9)	Supplementation: folic acid 5 mg/kg/day for 8 weeks	Six-week-old male Sprague–Dawley rats with HFD-induced steatohepatitis	16S rRNA sequencing; qPCR	Increased levels of Bacteroidetes, *Pseudomonadaceae*, and *Leptotrichiaceae*	No effect on body weight; reduced hepatic lipid accumulation, ballooning degeneration, inflammatory infiltration, and severe fibrosis (*p* < 0.05); reduced expressions of αSMA, TGF-β1, Col1a1, Col2a1, and Col3a1 (*p* < 0.05); significant reduction in ALT, AST, FBG, TG, TC, LDL, TBA, and Hcy (*p* < 0.05); no significant change in HDL; downregulation of mRNA expression levels of *SREBP1c*, *SCD*, *ACACA*, and *FASN* (*p* < 0.01) and upregulation of *PPARγ*, *ACADL*, *FABP1*, *CPT1α*, and *FATP2* (*p* < 0.01); decreased expression of pro-inflammatory proteins TNF-α, IL-6, IL-1β, and CCR2 (*p* < 0.05)	[65]
	Supplementation: 84 µg/kg folic acid per day for 8 weeks	2-month-old Sprague–Dawley male rats with hyperuricemia	16S rRNA gene sequencing	Increased abundance of Actinobacteria, *Lactobacillus*, *Bacteroides*, *Collinsella*, and *Blautia*; decreased abundance of *Clostridium*, *Romboutsia*, *Norank-f-Lachnospiraceae*, and *Ruminococcus*	Decreased levels of serum uric acid levels (*p* < 0.01), and adenosine deaminase and xanthine oxidase (*p* < 0.05)	[66]
	Supplementation: folic acid 5 mg/kg for 25 weeks	3- to 4-week-old male C57BL/6J conventional (CV) and germ-free (GF) mice with HFD-induced obesity	16S rRNA gene sequencing; qPCR; HPLC-MS/MS	Decreased abundance of unclassified_*f_Lachnospiraceae*, un classified_*g_norank_f_Oscillospiraceae*, unclassified_*g_Lachnospiraceae*_NK4A136_group, uncultured_bacterium_*g_norank_f_Oscillospiraceae*, uncultured_bacterium_*g_norank_f_Lachnospiraceae*, uncultured_bacterium_*g_Oscillibacter*, uncultured_bacterium_*g_Bilophila*, uncultured_bacterium_*g_Roseburia*, uncultured_bacterium_g_UCG-009, and uncultured_bacterium_*g_Tuzzerella*; increased abundance of uncultured_bacterium_*g_norank_f_Muribaculaceae*, *Ileibacterium*_*valens*, *Akkermansia_muciniphila*, uncultured_bacterium_*g_Dubosiella*, uncultured_bacterium_*g_Coriobacteriaceae*_UCG-002, unclassified_*g_norank_f_norank*_o_Clostridia_UCG-014, uncultured_Clostridiales_bacterium_*g_norank_f_Oscillospiraceae*, and unclassified_*g_Rikenellaceae*_RC9_gut_group; reduction in fecal dysbiosis	Reduced weight gain; reduced plasma level of BCAAs (valine, isoleucine, and leucine, *p* < 0.05); mRNA levels of *Bcat2*, *Bckdha*, and/or *Ppm1k* were increased in adipose tissues but decreased in the liver (*p* < 0.05); increased mRNA levels of mitochondrial biogenesis genes: *Pgc-1a*, *Cox1*, *Nd1* or *Nd6* (*p* < 0.05);	[67]
	Supplementation: 5.0 mg/kg/day folic acid for 10 weeks	7-week-old male C57BL/6J mice with alcohol-induced liver damage	16S rRNA gene sequencing	Increased relative abundance of Verrucomicrobiota and Proteobacteria, *Lachnospiraceae_NK4A136_group*, and *Akkermansia*	Reduced levels of ALT, AST, TG, and LPS, and inflammatory cytokines, IL-1β, IL-6, and TNF-α while tight junction proteins ZO-1, claudin 1, and occludin significantly increased (*p* < 0.05); reduced expression of TLR4, MyD88, IRAK1, TRAF6, p-IκBα/IκBα, and NF-κB (*p* < 0.05)	[68]
	Supplementation: three doses of folic acid (10, 80, or 150 μg/kg/day) for 7 days	6-week-old male Sprague–Dawley (SD) rats with chronic visceral hyperalgesia/IBD	16S rRNA gene sequencing	For all doses: reduced I number without affecting α-diversity (*p* > 0.05); decreased abundance of Clostridiales (*p* < 0.05); reduced H_2_S concentration (80 μg/kg; *p* < 0.001)	Attenuation of chronic visceral pain; frequency of sEPSCs of neurons in the spinal dorsal horn significantly reduced (*p* < 0.05); overall reduction in spontaneous glutamatergic synaptic activity of SG neurons	[69]
	Supplementation: 2.5 mg/kg/day and 5 mg/kg/day folic acid (L-FA) for 10 weeks	7-week-old male C57BL/6J mice with hyperuricemia	16S rRNA gene sequencing	Decreased relative abundance of Firmicutes while Bacteroidetes was not significantly changed; increased *Lactobacillus* and *Lactococcus* (*p* < 0.05)	High-dose FA restored expression of GLUT9, ABCG2, α-SMA, and E-cad (*p* < 0.05) and increased protein expression levels of Claudin-1, Occludin, ZO-1, and SCFAs (acetic acid and propionic acid) (*p* < 0.05); reversed elevated levels of TNFα, IL-1β, IL-6, TLR4, MYD88, and p-IκB and LPS (*p* < 0.05)	[70]
Serine	Supplementation: 40 mg/kg body weight, once orally for 7 days	9-week-old male C57BL/6 mice with acute colitis	16S rRNA gene sequencing	No significant difference in alpha diversity; increased relative abundance of Firmicutes, *Clostridia*, and Bacteroidia	Reversed weight loss; no significant change in colon length, colon weight, and length/weight ratio of colon but significant decrease in disease activity index (*p* > 0.05); increased levels of IgA, IgG, and IgM; decreased IL-1β, IL-6, TNF-α, MPO, and EPO levels (*p* > 0.05)	[71]
	Deficiency: L-serine deficient diet (ΔSer, TD.140546) for 3 days	6- to 12-week-old female and male SPF C57BL/6 mice, and GF Swiss Webster mice with IBD	qPCR; 16S rRNA gene sequencing	Increased relative abundances of *Verrucomicrobiaceae (and A. muciniphila)* and *Enterobacteriaceae (*and *E. coli)*, while *Sutterellaceae* and *Porphyromonadaceae* were decreased	Reduced body weight and increased colon inflammation; degradation of mucus layer; increased intestinal permeability	[72]
	Deficiency: serine- and glycine-deficient (SGD) diet for 2 months	9-week-old male C57BL/6J mice with inflammation and oxidative stress	16S rRNA gene sequencing; RT-qPCR	Significant decrease in Firmicutes to Bacteroidetes ratio, and relative abundance of *Clostridium* XIVa was further decreased	Increased accumulation of advanced glycation end products, and MDA; high serum levels of TNF-α, IL-1β, and IL-6; levels of SOD, CAT, GSH-Px, and GSH were significantly reduced (*p* < 0.05); increased mRNA levels of *TNF-α*, *IL-1β*, and *IL-6*, while *Cat*, *Sod1*, *Sod2*, and *Gpx1* were decreased (*p* < 0.05); a significant decrease in butyric acid but not change in acetate or propionate levels (*p* < 0.05); a significant decrease in mRNA expression of *Slc16a3*, *Slc16a7*, and *Gpr109a* (*p* < 0.05); increased pNF-kB, and decreased pAMPK (*p* < 0.05)	[73]
Methionine	Dietary methionine restriction (MR) at 0% or 80% for 16 weeks	8-month-old male C57BL/6J mice with age-related or HFD-induced diseases	16S rRNA gene sequencing; RT-qPCR	Increased relative abundance of Firmicutes and decreased Proteobacteria and Verrucomicrobia by either MR0 or MR80; MR80 showed an increased relative abundance of *Bacteroides*, *Faecalibaculum*, *Corynebacterium*, and *Roseburia* while *Desulfovibrio*, *Lachnospiraceae*, *Akkermansia*, *Lachnoclostridium*, *Oscillibacter*, *Ruminiclostridium*, and *Escherichia−Shigella* decreased (*p* < 0.05)	MR80 reduced body weight and IWAT (*p* < 0.05); increased levels of acetic, butyric, and propionic acid (*p* < 0.05); reduced serum LPS and LBP (*p* < 0.05); reduced serum and ileal TNF-α, IL-6, IL-1β, and IL-10 (*p* < 0.05); increased claudin-3, occludin, and ZO-1 (*p < 0.*05)	[74]
	Dietary methionine restriction (MRD) at 0.86% for 22 weeks	4-week-old male C57BL/6J mice with HFD-induced obesity	16S rRNA gene sequencing; qPCR; ^1^H NMR; GC-MS-QP2010	Significant increase in the relative abundance of Firmicutes (*p* < 0.05), and Firmicutes/Bacteroides ratio (*p* < 0.01); reduction in Verrucomicrobia (*p* < 0.05); MRD showed enrichment in *Allobaculum*, *Bacteroides*, *Oscillospira*, *Bifidobacterium*, *Sutterella*, *Roseburia*, *Lactobacillus*, *Bilophila*, and *Stenotrophomonas* at the genus level; increased relative abundance of *Bacteroides*, *Bifidobacterium*, *Oscillospira*, *Ruminococcus*, *Coprococcus*, *Corynebacterium*, *Lactobacillus*, and *Roseburia* while *Akkermansia* and *Desulfovibrio* were reduced (*p <* 0.05)	Significant decrease in body weight, food intake, blood glucose, plasma TG, TC, FFA, and LDL-c while HDL-c was increased (*p* < 0.05); increased levels of SCFAs such as formate, acetate, propionate, butyrate, lactate, pyruvate, succinate, α-keto-β-methyl-valerate, α-ketoisovalerate; increase in amino acid-related metabolites, 4-hydroxyphenylactate, and histidine and decreased levels of isoleucine, valine, glycine, tyrosine, urocanate, methionine; bile acids-related metabolites such as bile acids and taurocholic acid were increased while taurine was decreased; carbohydrate-related metabolites including β-glucose, α-glucose, α-xylose, and α-galactose were decreased; other metabolites like xanthine, trimethylamine, and ethanol were also reduced; (*p* < 0.05); significant increase in ileum and colon GSH-Px, GSH/GSSG, and T-AOC levels (*p* < 0.05); decreased plasma LPS, LBP, TNF-α, IL-6, colonic mRNA expression of *CD14* and *TLR4*, and *LBP*, *MyD88*, *NF-κB*, *TNF-α*, and *IL-6* in colon and ileum (*p* < 0.05); increased colonic and ileal tight junction proteins claudin-3, ZO-1, and occludin mRNA expression (*p* < 0.05)	
Tryptophan	Dietary tryptophan restriction: 10% (10TRP), 40% (40TRP), and 70% (70TRP) for 21 days	3-week-old male obesity-prone rats (OP-CD, Strain 463)	qPCR; TD-NMR;	No significant change with 70TRP; 40TRP and 10TRP showed a reduction in gene copies of *Enterobacteriaceae* and *Lactobacillus* but increased *Roseburia;* all TRP diets reduced 16S rRNA gene copies of *Bacteroides* and *Clostridium coccoides;* tryptophan restriction did not affect *Bifidobacterium* spp., *Clostridium leptum*, *and Clostridium perfringens*	Decreased food intake, energy expenditure, body weight, fat mass, lean mass, fasting blood glucose, plasma insulin, leptin, C-peptide and increased plasma glucagon, GLP-1, QUICKI, and pancreatic polypeptide by 40TRP and 10TRP; 10TRP increased plasma amylin and ghrelin; 70TRP and 40TRP increased plasma PYY without plasma GIP change (*p* < 0.05)	[75]
	Dietary tryptophan restriction deficient: 0.1%, recommended: 0.2%, and high: 1.25% diets for 8 weeks	20-month-old male C57BL/6 mice with systemic inflammation and gut dysbiosis	16S rRNA gene sequencing	High-dose TRP restored a relative abundance of Proteobacteria, Deferribacteres, *Mucispirillum*, and *Lachnospiraceae* that were reduced by low TRP while the increased abundance of *Acetatifactor*, *Enterorhabdus*, and *Adlercreutzi* was decreased with high TRP	Elevated serum levels of IL-6, IL-1a, and IL-17a, and decreased IL-27 by TRP-deficient diet compared to TRP-rich diets (*p* < 0.05)	[76]
	Dietary tryptophan supplementation: 200 mg/kg tryptophan for 2 weeks	6- to 8-week-old male BALB/c mice with Intestinal inflammation	16S rRNA sequencing; RT-qPCR; qPCR	Reduced Firmicutes/Bacteroidetes ratio; a high proportion of Clostridiales_un-Classified, *Acetivibrio*, *Cetobacterium*, and low *Enterobacter*, *Pantoea*, and *Chromohalobacter*	Decreased expression of ileum IL-1β, TNF-α, IL-17, iNOS, and p-p65; elevated levels of IκBα; restoration of mRNA expression levels of α-defensin 5, Reg3b, Reg3g, mucin 2 and trefoil factor 3, and goblet cell differentiation factors Krüppel-like factor 4 and Ets-Domain Transcription Factor; reduced Beclin1 and LC3B-II: I ratio, p-AMPK, SIRT1, p-mTOR, and p-p70s6k and increased expression of SQSTM1 (*p* < 0.05)	[77]
	Dietary tryptophan restriction (95% reduction) for 12 weeks	16-week-old DNA-repair deficient, premature-aged mice (Ercc1^-^/^Δ7^; 20-wk life span); premature aging	16S rRNA gene sequencing	Increased microbial diversity and abundances of Bacteroidetes RC9 and Clostridiales; reduced proportion of *Alistipes* and *Akkermansia* spp. correlates with a decrease in the number of B-cell precursors; gut microbiota composition restored from aging phenotype to younger WT mice	Reduced body and spleen weight (*p* < 0.001); reduced frequencies of B lineage cells within total bone marrow cells and total B lineage cells; overall decrease in B-cell frequencies in mesenteric lymph node and spleen (*p* < 0.01); neutrophil numbers unaffected in bone marrow (*p* < 0.05); however, a significant reduction in splenic neutrophil and monocyte numbers (*p* < 0.05)	[78]
Iron	Iron deficiency and repletion: Fe-deficient diet for 24 days, repleted for 13 days with FeSO_4_ or electrolytic Fe at 10 and 20 mg Fe kg/diet; a total of 37 days	21-day-old male Sprague–Dawley rats	TGGE and qPCR	Fe-deficient group: decreased *Bacteroides* spp. and *Roseburia* spp. while *Enterobacteriaceae* increased; Fe-repletion reversed the trend and *Lactobacillus/Pediococcus/Leuconostoc* spp. significantly decreased to baseline levels (*p* < 0.05)	Fe deficiency resulted in reduced weight gain and food intake, cecal butyrate (−87%) and propionate (−72%) levels (*p* < 0.05); repletion restored and increased cecal butyrate, and neutrophil infiltration in colonic mucosa (*p* < 0.01)	[79]
	Iron depletion for 12 weeks (2·9 mg Fe/kg diet) and repletion for 4 weeks (35 and 70 mg Fe/kg diet)	8-week-old female Fischer 344 rats	qPCR and pyrosequencing; HPLC	Low relative abundance of *Bilophila* spp. and *Coprococcus* spp.; 35 ppm Fe-supplemented rats have higher *Bacteroides* spp., *Clostridium* cluster IV, *F. prausnitzii*, *E. hallii*, and sulfate-reducing bacteria than Fe-deficient rats; no significant change between 70 ppm supplementation and Fe-deficient rats	Significant increase in acetate and propionate concentrations, and Fermentative metabolites by 35 and 70 ppm Fe-supplementation with butyrate increasing significantly by 35 ppm	[80]
	Iron-deficient (<10 ppm iron/kg diet); control (35 ppm iron/kg diet); iron supplemented diets (200 ppm iron/kg) diet for 4 weeks	8- to 14-week-old WT 129S6/SvEV and colitis-susceptible interleukin-10-deficient (Il10^−/−^) mice; IBD associated intestinal microbiota	qPCR; 16S rRNA sequencing	The relative abundance of Proteobacteria, *Enterobacteriaceae*, and *Escherichia coli* was increased by low Fe and control compared to high Fe diet	High Fe showed reduced colitis based on clinical disease activity and histological inflammation; while high and low Fe decreased colonic and serum IL-12 p40 compared to control; modest reduction of colitis by high and low Fe	[81]
	Supplementation: chow diets containing 100, 200, or 400 ppm iron for 8 or 10 days	8- to 9-week-old Female C57BL/6 mice with DSS-induced colitis	16S rRNA gene sequencing	400 ppm reduced species richness, with a significant increase in Proteobacteria and Actinobacteria and a decrease in Bacteroidetes and Firmicutes	Significant reduction in body weight at 100 ppm (*p* < 0.01) while fecal calprotectin levels increased for 100 (*p* < 0.05) and 400 (*p* < 0.001) ppm at day 8 vs. 10; overall decrease in colitis by increasing dietary iron content	[82]
Copper	Supplementation: 1.6 (low), 6.0 (adequate), or 20 (high) ppm of copper for 4 weeks	Male weanling Sprague–Dawley rats with high-fructose-fed rats (NAFLD)	16S rRNA sequencing; qPCR	Reduced abundance of Verrucomicrobia; low-cupper diet exhibited more pronounced obesity phenotype featured by high Firmicutes/Bacteroidetes ratio, and decreased *Bacteroidaceae*, and *Bacteroides*; high-copper diet decreased *Bifidobacteriaceae* and *Bifidobacterium* but increased *Lactobacillaceae* and *Lactobacillus*, *Erysipelotrichaceae*, *Enterobacteriaceae.* Both low and high copper diets reduced *Akkermansia*	Increased levels of plasma AST, ALT, gut permeability, and steatosis; ileal Reg3b protein expression and IL-22 mRNA expression (*p* < 0.001); downregulation of protein expression of Claudin-1 and occludin by low- and high-copper diets (*p* < 0.001); elevated plasma endotoxin by low-copper diet (*p* < 0.0001)	[83]
	Supplementation: 5 mg/kg of copper for 90 days	8-week-old Kunming female mice	16S rRNA gene sequencing	Decreased abundance of *Rikenella*, *Jeotgailcoccus*, and *Staphylococcus*; increased abundance of *Corynebacterium* (*p* < 0.05)	Body weight decrease (*p* < 0.05); blunt intestinal villi, necrosis of enterocytes, severe atrophy of central lacteal, and decreased number of goblet cells	[84]
	Supplementation: 6, 120, and 240 mg/kg of copper fed for 8 weeks	21-day-old male Sprague–Dawley rats	Pyrosequencing; AAS	High copper dose (120 and 240 mg/kg) decreased abundance of *Christensenellaceae*, *Lachnospiraceae*, *Allobaculum*, *Flavonifractor*, *Oscillospira*, *Parabacteroides*-related OTUs, and *Blautia*-related OTUs; increased abundance of *Ruminococcaceae*, *Defluviitaleaceae*, *Peptococcaceae*, *Peptostreptococcaceae*, *Turicibacter*, *Coprococcus*, *Anaerotruncus*, *Peptococcus*, *Dorea*, *Rikenella*, *Barne*siella, *Bacteroides*, and *Alistipes*-related OTUs (*p* < 0.05)	No significant change in body weight and levels of inflammatory cytokines IL-1β, IL-6, and IL-8 (*p* < 0.05); TNF-α increased significantly with high copper level (*p* < 0.01)	[85]
	Supplementation: 0, 0.04, 0.20, or 1.00 mg/kg CuSO4 for 15 days	1-day-old Sprague–Dawley rats	16S rRNA gene sequencing; UPLC-Q-TOF	Dose-dependent impact on α- and β-diversity, and reduced Firmicutes/Bacteroidetes ratio; increasing copper levels increased *Treponema*_2 and *Erysipelatoclostridium* and decreased *Romboutsia*, *Chlamydia*, *Bifidobacterium*, and *Lactobacillus;* 0.04 mg/kg increased abundance of *Alloprevotella*, *Lachnospiraceae_NK4A136*, *Ruminiclostridium_5*, and *Ruminococcaceae_UCG-013* but declined with other copper levels	Increased serum ALT, AST, and ALP levels and decreased TP, ALB, and urea levels by 0.20 and 1.00 mg/kg dose (*p* < 0.05); no significant change in albumin, globulin, ratio of white balls, creatinine, and total cholesterol (*p* < 0.05). Additionally, 0.20 and 1.00 mg/kg doses showed inflammatory lesions, bile duct hyperplasia, and fatty degradation while only 1.00 mg/kg resulted in point necrosis; copper exposure reduced N-acetyl-D-glucosamine, xanthine, L-tyrosine, 2-phenylacetamide, phenylpyruvic acid, L-phenylalanine but increased 2-hydroxybenzaldehyde, 12-KETE, gamma-linolenic acid, and 20-hydroxyeicosatetraenoic acid (*p* < 0.05)	
Zinc	Supplementation: 0/29/1000 mg/kg of Zinc for 5 weeks	8- to 12-week-old C57BL/6 S100a9^−/−^ mice with Clostridium difficile	16S rRNA gene sequencing; qPCR; ICP-MS; LA-ICP-MS; MALDI IMS	High Zn diet showed decreased microbial diversity and OYUs of *Turicibacter* (OTU 2) and *Clostridium* (OTU 11) but increased *Enterococcus* (OTU 4) and *Clostridium* XI (OTU 3) (*p* < 0.001)	Increased level of IL-1β and MCP-1, and reduction in IL-6, IL-10, and IL-12 (p70) by high Zn diet (*p* < 0.05); decrease in epithelial damage and pseudomembrane formation	[86]
	Supplementation: 0/30/150/600 mg/kg of Zn for 4 and 8 weeks	3-week-old C57BL/6 mice	16S rRNA gene sequencing; GCMS	Increased abundance of Verrucomicrobia, *Akkermansia*, *Faecalibaculum*, *Helicobacter*, *Dubosiella*, *Caulobacter*, *Bradyrhizobium*, and *Ileibacterium* by high and excess Zn diet; decrease in *Romboutsia*, *Bacteroides*, *Lactobacillus*, and *Bifidobacterium* while Bacteroidetes/Firmicutes ratio increased (*p <* 0.05) with increasing Zn diet at 4 weeks; increased abundance of Actinobacteria, *Bifidobacterium*, and *Anaeroplasma* at 8 weeks in high Zn diets while Verrucomicrobia, *Intestinimonas*, and *Lactobacillus* decreased	Significant reduction in cecal metabolites and total SCFAs, acetic acid, butyric acid, isobutyric acid, isovaleric acid, and propionic acid by excess Zn at both 4 and 8 weeks (*p* < 0.05)	[87]
	Supplementation: 4 mg/kg zinc per day for 8 weeks	2-month-old Sprague–Dawley male rats with hyperuricemia	16S rRNA gene sequencing	Increased abundance of *Lactobacillus*, *Norank-f-Muribaculaceae*, and *Bacteroides*; decreased abundance of *Clostridium*, *Romboutsia*, *Blautia*, and *Norank-f-Lachnospiraceae*	Decreased levels of uric acid levels (*p* < 0.01), and adenosine deaminase and xanthine oxidase (*p* < 0.05)	[66]
Manganese	Supplementation: 100 ppm MnCl_2_ for 13 weeks	Male and female 7-week-old C57BL/6 mice	6S rRNA gene sequencings, metagenomics sequencing, and GC-MS metabolomics	Decrease α-diversity; increased relative abundance of Firmicutes and Tenericutes and decrease in Bacteroidetes and Verrucimirobia in males; decrease in Firmicutes and increased Verrucimirobia in females (*p* < 0.05)	Significant alteration in genes for tryptophan biosynthesis pathway in females with an increase in anthranilate phosphoribosyltransferase (*p* < 0.01), indole-3-glycerol phosphate synthase, and tryptophan synthase (α-chain) (*p* < 0.05), phenylalanine synthesis with increased biosynthetic aromatic amino acid aminotransferase (*p* < 0.001) and prephenate dehydratase (*p* < 0.05); in male, there is increase in tryptophan synthase (β-chain) (*p* < 0.001) and decrease in biosynthetic aromatic amino acid aminotransferase (*p* < 0.001) and prephenate dehydratase (*p* < 0.01); additional sex-specific alterations in GABA and putrescine biosynthesis genes, precursor of neurotransmitter synthesis, and LPS biosynthesis genes (*p* < 0.05); decrease in α-tocopherol and γ-tocopherol in both sexes	[88]
	Deficiency: <0.01 ppm Mn for 14 days	3–4 weeks C57BL/6 mice aged with DSS-induced colitis	16S rRNA gene sequencing; qPCR; ICP-MS	No significant fecal microbiota difference	Increased weight loss and 13% decrease in colon length (*p* < 0.05); a significant decrease in colon tight junction proteins Zo1, Zo2, Cldn2, Cldn3, and Ocln but not on Cldn4, 5, 7, 12, and 15 (*p* < 0.05); MnSOD enzyme activity reduced by 57%, (*p* < 0.01) with a significant increase in H_2_O_2_, 8-isoprostane (*p* < 0.05), and 8-hydroxy-2-deoxyguanosine (*p* < 0.01); decrease in chemokine (*Ccl2* and *Cxcl1*) expression; *Tnfα*, *Il6*, *Il1β*, and *Il10* showed no significant change (*p* < 0.05)	[89]

**Table 2:** Select randomized controlled trials on the physiological roles of kefir.

Research NCT/Population/Duration	Source of Intervention/Dose	Gut Microbiota Alteration	Physiological Effect	References
NCT03966846; 62 participants with metabolic syndrome (18–65 years); 12 weeks	DC1500I culture (Olsztyn, Poland); 180 mL of kefir daily; control: unfermented milk (180 mL)	Significant increase in Actinobacteria *(p =* 0.023); no significant change in Bacteroidetes, Proteobacteria, or Verrucomicrobia	Kefir intake resulted in decrease in serum TNF-α (*p* = 0.047; 40%), IL-6 (*p* = 0.01; 23.7%), IL-10 (*p <* 0.01 = 56.5%), IFN-γ (*p* < 0.01; 46.8%), homocysteine (*p* = 0.048; 4.3%), SBP (*p* < 0.01; 7%), and DBP (*p* = 0.04; 5.2%); no significant change in anthropometrical parameters (weight, BMI, WC, FM, FFM, TBW), glycemic parameters (glucose, insulin, HbA1c, HOMA-IR), and lipid profile (TC, HDL-c, ApoA1, ApoB, triglycerides); improvement in inflammatory markers	[96,97]
NCT01428999; 54 healthy adults (20–40 years); 3 weeks	AB-kefir and placebo products by SYNBIO TECH INC (Kaohsiung, Taiwan); AB-kefir group or placebo group; one sachet (2 g) of AB-kefir or placebo after meal twice a day	In males, a significant correlation was observed between (1) heightened abdominal bloating and a reduction in bifidobacteria (*p* = 0.022), (2) an elevated *E. coli* population (*p* = 0.080) and total aerobes (*p* = 0.096), (3) increased *bifidobacteria* and decreased total aerobes (*p* = 0.041) and (4) increased *E. coli* and increased total aerobes (*p* = 0.008). In females, there was an increase in total anaerobes (0.49 log CFU/g; *p* = 0.038) and total gut microbial counts (0.45 log CFU/g; *p* = 0.049)	Kefir consumption showed a reduction in symptoms of abdominal pain, bloating (*p* = 0.014), and appetite (*p* = 0.041); general improvement in gastrointestinal functions	[98]
NCT02849275; 26 healthy adults (25–45 years); 4 weeks	Dairy-fermented beverage (25–30 × 10^9^ CFU of active kefir cultures); 8 oz of a dairy-based fermented beverage or control (8 oz non-fermented beverage)	Increased abundance of *Lactobacillus* (*p* < 0.01)	Significant improvement in performance on two metrics of relational memory, misplacement (*p* = 0.04) and object–location binding (*p* = 0.03); although no observed correlations between *Lactobacillus* abundance and memory performance; improvement in hippocampal-dependent relational memory	[99]
45 patients with IBD (UC: 19–68 years, CD: 24–65 years); 4 weeks	Kefir fermented and produced under anaerobic conditions (Kefir culture: 2.0 × 10^10^ CFU/mL viable Lactobacillus bacteria); 400 mL/day kefir; control group: no treatment	Kefir significantly increased fecal *Lactobacillus* in CD compared to the control group (*p* < 0.024); no significant change in UC. UC and CD showed increased *Lactobacillus* bacterial fecal load (*p =* 0.001 and *p* = 0.005) after kefir intake (week 4)	Significant reduction in bloating scores (*p* = 0.012) and feeling good scores significantly increased (*p* = 0.032) in CD	[100]

Abbreviations: WC, waist circumference; ApoB, apolipoprotein B; SBP, systolic blood pressure; TBW, total body water; DBP, diastolic blood pressure; FFM, fat-free mass; BMI, body mass index; FM, fat mass; ApoA1, apolipoprotein A1; HDL-c, high-density cholesterol; HOMA-IR, homeostasis model assessment for insulin resistance; IFN-γ, interferon-gamma; IL, interleukin; LDL-c, low-density cholesterol; TC, total cholesterol; TNF-α, tumor necrosis factor-α; HbA1c, hemoglobin A1C; CRP, C-reactive protein; sICAM-1, secretory intercellular adhesion molecule 1; sVCAM-1, secretory vascular cell adhesion molecule 1; UC, ulcerative colitis; CD, Crohn’s disease; TUNEL, terminal deoxynucleotidyl transferase dUTP nick-end labeling; DMH, 1,2-dimethylhydrazine.

## Abbreviations

WC, waist circumference; ApoB, apolipoprotein B; SBP, systolic blood pressure; TBW, total body water; DBP, diastolic blood pressure; FFM, fat-free mass; BMI, body mass index; FM, fat mass; ApoA1, apolipoprotein A1; HDL-c, high-density cholesterol; HOMA-IR, homeostasis model assessment for insulin resistance; IFN-γ, interferon-gamma; IL, interleukin; LDL-c, low-density cholesterol; TC, total cholesterol; TNF-α, tumor necrosis factor-α; HbA1c, hemoglobin A1C; CRP, C-reactive protein; sICAM-1, secretory intercellular adhesion molecule 1; sVCAM-1, secretory vascular cell adhesion molecule 1; UC, ulcerative colitis; CD, Crohn’s disease; TUNEL, terminal deoxynucleotidyl transferase dUTP nick-end labeling; DMH, 1,2-dimethylhydrazine; BCFA, branched short chain fatty acid; 11β-Hsd1, 11-β-hydroxy-steroid-dehydrogenase 1; BCAA, branched chain amino acids; A β, amyloid-β peptides; BACE1, beta-site APP-cleaving enzyme 1; BDNF, brain-derived neurotrophic factor; GABA, γ-aminobutyric acid; APP/PS1, amyloid precursor protein/presenilin 1; P-tau, phosphorylated Tau; RXRs, retinoid X receptors; RALDH1, retinaldehyde dehydrogenase 1; RAR, retinoic acid receptors; CYP26B1, cytochrome P450 family 26 subfamily B member 1; DAO, diamine oxidase; αSMA, α-smooth muscle actin; Col1a1, collagen type I alpha 1; Col2a1, collagen type II alpha 1; Col3a1, collagen type III alpha 1; FATP2, fatty acid transport protein 2; ACADL, long-chain specific acyl-CoA dehydrogenase; PPARγ, peroxisome proliferator-activated receptor gamma; TG, triglycerides; Hcy, homocysteine; TBA, total bile acids; TC, total serum cholesterol; CPT1α, carnitine palmitoyltransferase 1A; AST, aspartate aminotransferase; ALT, alanine aminotransferase; FBG, fast blood glucose; HDL, high-density lipoprotein; LDL, low-density lipoprotein; CCR2, chemokine receptor C-C chemokine receptor type 2; ACACA, acetyl-CoA carboxylase; FASN, fatty acid synthase; SREBP1c, sterol regulatory element binding transcription protein 1c; SCD, stearoyl CoA desaturase; FABP1, fatty acid-binding protein 1; TLR4, toll-like receptor 4; MyD88, myeloid differentiation primary response protein 88; IRAK1, interleukin 1 receptor-associated kinase 1; TRAF6, tumor necrosis factor receptor associated factor 6; IκBα, nuclear factor of kappa light polypeptide gene enhancer in B-cells inhibitor, alpha; sEPSCs, spontaneous excitatory post-synaptic currents; Bcat2, branched-chain amino acid transaminase 2; Bckdha, branched-chain keto acid dehydrogenase E1 subunit alpha; Ppm1k, protein phosphatase 1K; Pgc-1a, Ppar-g coactivator 1a; Cox1, cytochrome c oxidase subunit 1; Nd1, NADH dehydrogenase subunit 1; Nd6, NADH dehydrogenase subunit 6; T-AOC, total antioxidant capacity; QUICKI, quantitative insulin sensitivity check index.
